# Are patients open to elective re-sampling of their glioblastoma? A new way of assessing treatment innovations

**DOI:** 10.1007/s00701-014-2189-3

**Published:** 2014-08-02

**Authors:** Taskia Mir, Peter Dirks, Warren P. Mason, Mark Bernstein

**Affiliations:** 1 Division of Neurosurgery, Toronto Western Hospital, University of Toronto, West Wing, 4th Floor Rm 4 W448, 399 Bathurst St., Toronto, ON M5T2S8 Canada; 2 The Hospital for Sick Children, 444 University Ave., Toronto, ON M5G1X8 Canada; 3 Princess Margaret Cancer Center, 18th Floor Rm. 717, 610 University Ave., Toronto, ON M5G2M9 Canada

**Keywords:** Asymptomatic relapse, Ethics, Glioblastoma, Innovation, Qualitative research, Re-sampling

## Abstract

**Background:**

This is a qualitative study designed to examine patient acceptability of re-sampling surgery for glioblastoma multiforme (GBM) electively post-therapy or at asymptomatic relapse.

**Methods:**

Thirty patients were selected using the convenience sampling method and interviewed. Patients were presented with hypothetical scenarios including a scenario in which the surgery was offered to them routinely and a scenario in which the surgery was in a clinical trial.

**Results:**

The results of the study suggest that about two thirds of the patients offered the surgery on a routine basis would be interested, and half of the patients would agree to the surgery as part of a clinical trial. Several overarching themes emerged, some of which include: patients expressed ethical concerns about offering financial incentives or compensation to the patients or surgeons involved in the study; patients were concerned about appropriate communication and full disclosure about the procedures involved, the legalities of tumor ownership and the use of the tumor post-surgery; patients may feel alone or vulnerable when they are approached about the surgery; patients and their families expressed immense trust in their surgeon and indicated that this trust is a major determinant of their agreeing to surgery.

**Conclusion:**

The overall positive response to re-sampling surgery suggests that this procedure, if designed with all the ethical concerns attended to, would be welcomed by most patients. This approach of asking patients beforehand if a treatment innovation is acceptable would appear to be more practical and ethically desirable than previous practice.

## Introduction

The treatment of glioblastoma multiforme (GBM) typically involves a multimodal intervention consisting of a craniotomy for tumor resection and radiotherapy with concomitant and adjuvant chemotherapy with temozolomide (TMZ). Despite the currently available regimens, therapeutic resistance remains one of the major challenges [4]. It would be advantageous for neuro-oncologists to explore other feasible personalized treatment options that may enable clinicians to improve patient outcomes.

There is emerging evidence that recurrent neoplasia, as a result of clonal evolution, is often distinct genetically from the disease at diagnosis, and we cannot expect treatment decisions to be made upon the initial diagnostic sample to guide effective therapy throughout the course of the disease. In particular, use of the standard of care agent, temozolomide, is associated with a marked increase in the numbers of mutational events, suggesting that the very therapy that contributes to improved survival also leads to a mutationally more complex disease at progression. In order to understand the key genomic changes and possible distinct therapeutic targets at the time of recurrence, we must obtain a sample of the patient’s “new” disease, as the original specimen is genetically out of date. This is routine for patients with hematological malignancies where the risk of bone marrow biopsy is low, but for glioblastoma patients this would mean another craniotomy and/or needle biopsy some time after treatment initiation and/or at asymptomatic relapse [21]. This might be particularly relevant for glioblastoma with recent demonstration of several distinct molecular subtypes [22]. From a clinical perspective, setting a standard of tumor re-sampling as an essential measure of GBM evolution in every patient would be paradigm shifting. Even though current technical advances in neurosurgery make tumor sampling acceptably low risk, it is imperative to acknowledge not only the prospective long-term benefits of undergoing tumor re-sampling, but also the potential disadvantages [14].

Patients undergoing a repeat craniotomy for tumor re-sampling post-treatment initiation and/or at asymptomatic relapse may encounter the following problems: increased risk of infection [1, 6, 8, 12, 15]; increased risk of neurological deficits [5, 7, 10, 18]; increased risk of psychological complications such as depression [9, 16, 17]; additional normal living time lost [13]; and a substantial increase in GBM treatment cost [20].

Planned repeat surgery may have potential survival benefits, and repeat sampling could facilitate a deeper understanding of glioblastoma evolution and help to identify new potential treatment targets or readjust prognosis. Although it is hypothesized that this strategy, in the long run, will be essential to enable appropriate modification of patient treatment to genetic/epigenetic clonal evolution, it is important that we understand the short-term implications as well. Because we are in the preliminary stages of investigating this novel concept, there may exist ethical and other challenges to performing a repeat surgery on malignant brain tumor patients with no clinical indications, as we currently define them.

In order to properly establish the parameters that will enable clinicians to re-sample glioblastoma post-treatment initiation and/or at asymptomatic recurrence, it is essential to involve patients in helping us create ethically appropriate standards for tumor re-sampling. We thus conducted a study using qualitative methodology to gain insight into brain tumor patients’ views about re-operation for molecular diagnosis. The results of this study may determine whether there is a “marketplace” for the use of routine post-treatment sampling of glioblastomas, and more generically this approach may represent a new paradigm for assessing whether patients are open to new treatments before they are imposed upon them.

## Methods

### Study design

This study was conducted using qualitative research methodology. Patients seen in consultation for malignant glioma were interviewed using a semi-structured, open-ended questionnaire (Appendix). All patients had at least one surgery, and a number had two. Patients’ perceptions regarding re-sampling of brain tumor post-therapy were explored in detail.

### Setting and participants

Participants were ambulatory patients recruited from a Neuro-Oncology Clinic in a tertiary care academic hospital. This clinic sees patients from three adult hospitals with neurosurgical services staffed by a total of about 25 neurosurgeons. Patients who were: (1) <18 years of age, (2) aphasic, or (3) not sufficiently cognitively intact and/or proficient in English were excluded from the study.

### Sample size

Thirty interviews were conducted on eligible and consented patients. This sample size was selected because it was anticipated that this number would be deemed sufficient in attaining thematic saturation. Saturation in qualitative research describes the point at which successive interviews will not yield any new concepts or ideas beyond ones that have already arisen [19].

### Data collection

Open-ended, face-to-face interviews were conducted with each participant by a single investigator with no clinical relationship with the patients. A semi-structured interview guide was used (see Appendix). Themes were freely explored as they emerged. All interviews were digitally audio-recorded and transcribed. Demographic parameters including age, sex, religion, marital status, education, and occupation were collected, as well as clinical data such as specific diagnosis and date(s) of surgery.

### Data analysis

Interviews were examined through modified thematic analysis involving open and axial coding [19]. Open coding involves breaking down information into common groupings based on shared ideas, whereas axial coding involves organizing information according to overarching themes.

### Research ethics

Participation was entirely voluntary, and informed consent was obtained from all participants. Both the confidentiality of patients and their freedom to withdraw from the study at any time was explained and maintained. The study was passed by the Research Ethics Board of the University Health Network.

## Results

Demographic information on the 30 interviewed patients is included in Table 1. Their responses were analyzed, and the resulting data were organized into several themes. Table 2 presents some of the most commonly asked questions by patients. The themes that emerged are presented below.

**Table 1:** Patient demographic data

Characteristics	Value
Age (years)	
Range	23–73
Mean	46
Sex	
Male	17
Female	13
Number of surgeries	
Single	26
Multiple	4
Tumor location	
Frontal	8
Parietal	3
Temporal	5
Occipital	2
Brainstem/ventricular	1
Multifocal	6
Complications during surgery	
Serious	3
Moderate	1
Mild	1
None	25
Resection	
Total	8
Partial	19
Postoperative complications	
Headaches	9
Fatigue	8
Function loss	4
Vision deficits	3
Memory loss	2
Swelling	1
Nausea	2
Seizures	2
None	12
Length of hospital stay	
Range	0–21
Mean	4.6
Religion	
Christian	11
Muslim	1
Jewish	1
Sikh	1
Other	4
Atheist	11
Undeclared	1
Education	
High school	5
College diploma	2
University/college degree	19
Undeclared	4
Occupation	
Engineering	2
Service industry	15
Administration	2
Educator	2
Healthcare	2
Law	1
Retired	3
None	2
Undeclared	1
Marital status	
Single	7
Married	17
Other	4
Undeclared	2

**Table 2:** Most common questions patients asked

○ “How much are you cutting? What would the surgery actually look like?”	
○ “What is the likelihood of my quality of life staying the same?”	
○ “What is the research supporting this biopsy?”	
○ “What happens to the tumor after it is taken out?”	
○ “What are the chances of me experiencing a complication during surgery and/or dying during surgery?”	
○ “Is this the best option right now? Are there better options? Are we being open minded?”	
○ “What are we hoping to achieve?”	
○ “What is the healing time needed?”	
○ “Would you have the surgery and would you suggest the surgery to your children?”	

### The major reason that patients were willing to undergo the surgery is their surgeon

All of the patients indicated that they would only agree to the surgery if they have faith in the skill level, experience, and credentials of the surgeon. Many patients specified that they would only undergo the surgery again if they had the same surgeon as for their first surgery. Several patients also required trust in the intentions of the surgeon. Patients feel that an ethical concern could arise if the surgeon is in a position to exploit a patient’s trust in order to increase their own credentials and/or acquire a new publication. If they feel that the surgeon has their best interests at heart, they are more comfortable going forward with the surgery. Many patients would ask the surgeon whether they would suggest the surgery to their own family, accepting their response as a strong measure of the validity of the surgery.

### Time is an important but variable factor for patients, depending mostly on patients’ requirement to take time off work, time for recovery, and time to reintegrate into their normal life

For patients who have to travel distances to come to the hospital, and/or need to miss work or family time both in the hospital and during the recovery phase, lost time was of variable but often significant importance. Some patients felt that the surgery was worth the extra time expenditure, while others would find it to be a deterrent. Patients who are making goals for the future and are asymptomatic are less interested in surgery regardless of their prognosis.

### The wish to be altruistic is very strong—this includes patients who would not agree to the routine surgery but would agree to participate in a trial

Some patients indicated that they would not be interested in the operation if it were offered to them off study, fearing the possible side effects and negative impact on their quality of life. In spite of having such reservations, patients agreed to a clinical trial for such a procedure. They indicated that they believe in the benefits of the procedure even though they might not want it for themselves. They hope that their participation would result in better treatment for others.

### Experience during the first surgery and/or with previous clinical trials is a major determinant of a patient’s willingness to undergo a second surgery

Patients who had experienced complications during their first surgery were reluctant to consider repeat surgery. Every patient who agreed to the second surgery had a relatively quick recovery and minimal side effects. All patients except two said prior complications would change their willingness to agree dramatically, explaining that complications are a matter of luck and can happen to anyone at any time. Feelings of trust in clinical trials are elevated by positive previous experiences with clinical trials.

### Second surgery within 3 months of the first may be too overwhelming for many patients

Some patients were willing and ready, while others stated that they were just starting to come to terms with their diagnosis at that time, and they are not ready to be approached about a second surgery. The state of patient’s emotional well-being determines their willingness to consider the surgery. Patients mentioned the role of hope in their emotional well-being. A second surgery and/or asymptomatic relapse reminds them that there is a serious problem and reduces the feeling of hope. Two patients would agree to the surgery because they felt that their lives are effectively over, and wish to be altruistic and “volunteer their brains.” This raises ethical issues because some patients’ hopelessness may be inadvertently taken advantage of.

### No matter what decisions they make, patients do not want to sacrifice quality of life

Patients dread the possibility of their surgery causing them to lose functionality and become dependent on their families. Becoming a physical and financial burden provokes anxiety and reluctance to agree to surgery. Patients give more weight to family opinions in the case of the trial than they do in the case of a surgery that is proven.

### Overall, patients were generally interested in this surgery

Of the 30 patients interviewed during this study, 22 would be interested in a routine GBM re-sampling biopsy should it be offered, provided all ethical and patient comfort grounds discussed above are addressed. Eight patients would not be interested in the surgery for personal comfort purposes, although all eight expressed support for the research. Sixteen patients would agree to participate in a clinical trial for such a procedure given that all ethical issues are addressed. Two more patients indicated that they might be interested in a clinical trial; however, it would depend strongly on their emotional and physical stability at the time. Twelve patients would not be interested in participating in a clinical trial at all.

### Some patients expect financial compensation if they have to take time off work or travel for the treatment, while others felt financial incentives might increase patient unintentional participation

Financial incentive to the patient was a major ethical concern, as it would increase the possibility of agreement to the surgery. Conversely, patients felt it was unethical to expect a patient to incur an extra financial burden to undergo a trial surgery at a time when many are already financially strained. Patients also expressed concerns about financial incentives to the surgeon and/or researcher.

### Complete communication as well as disclosure prior to surgery was a major concern for most of the interviewed patients

Patients expressed concerns about incomplete explanation of the procedures. Patients would prefer to have a clearer picture of the procedure before agreeing to it. Some patients are interested in seeing the specific research behind the experimental surgery. Patients also indicated that they would appreciate this information being provided during the first diagnosis or earlier in their treatment plan, allowing them to psychologically prepare for being asked about a second invasive procedure. Some patients are interested in detailed explanation of where/how their tumor will be handled and the legalities of ownership of the tumor after resection.

### The appointment where the patient is told about the surgery is a very emotionally draining time, which may impact their decision-making

Patients are often too shocked to take in information about the procedures. Patients who feel that they have no other way to survive may consent to the surgery because they are so worried about their diagnosis. In the case of an asymptomatic relapse, the fact that there is a relapse of tumor in their brain is the main thing that patients hear. They may not necessarily comprehend the implications or prognostic significance of an asymptomatic tumor. Shocked and desperate, patients feel that they often agree to procedures without due analysis; they need time to consider their options and choose wisely, such that they do not regret their decisions post-surgery. In the interviews performed, there was a clear trend where patients who initially agreed to the surgery and the trial very enthusiastically often became less enthusiastic as the interview progressed and they were asked more specific questions about their feelings. Some patients require more time to go home and think about the situation in a more comfortable setting.

## Discussion

When considering any surgery, patients must contemplate several facts and weigh the pros and cons of the surgery before making a decision. Surgery for re-sampling GBM represents a surgical innovation [2]. Some factors patients considered regarding the acceptance of this innovation include: trust in their surgeon, expenditure of time, financial burden to themselves and their family, wish to be altruistic, psychological and physical well-being, and their experience during the first surgery. The participants also highlighted numerous ethical concerns that they or other potential patients may have, and that may result in patient reluctance to participate, including financial incentives, lack of communication, exploitation of patient vulnerability, and surgeon transparency.

From the results of this study, trust in one’s surgeon is paramount and arguably the single most important factor in a patient accepting such a surgery. The importance of trust has not surprisingly been reported to be very important for patients participating in many other qualitative studies [3, 11]. Because this surgery would be a second or third surgery, presumably the patient would have earned trust in his/her surgeon, since they got the patient through it the first time. Some concepts, such as re-sampling, are nuanced and complex, while one’s trust in another human being, such as their surgeon, is basic and may trump other factors. The process will also be helped by the neuro-oncologist and radiation oncologist being on board with the plan; these members of the team will obviously have seen and gotten to know every patient considered for re-sampling surgery.

It is important to ensure that the consent discussions and documentation contain clear and understandable details about the surgery. Some patients are interested in exactly what will happen to their tumor post-surgery. This may be addressed by including a section in the consent documents that specifies who has rights and access to the tumor after resection and what will happen to the extracted components.

Patients perceived a major ethical concern with the fact that they often feel vulnerable and hopeless when they are told about any surgery. At such a time, patients would appreciate having a family member present to help interpret the situation and to give support. It may also be beneficial to allow the patient and their family whatever time they need to consider all aspects of the surgery. Surgeons are used to immediate results including getting consent the day surgery is suggested, but while this is efficient, it may not work well for some. Patients who are initially against the surgery may decide otherwise once they feel more comfortable and familiar with the procedure.

Another important consideration for the patients is the cost. Some patients indicated that they are concerned about travel costs, costs of parking, costs of staying in a hotel if they are not from the city, lost earnings from taking time off of work, and other associated costs. Patients may appreciate some compensation for such expenditures should they participate. A major ethical concern that arises from monetary compensation is the distinction between compensation and monetary incentives [23]. Patients felt an ethical dilemma may emerge in that financial gain from participating in a trial may cloud their judgment. Such factors should be considered when designing the study as it might be onerous to request that patients pay to travel to the hospital and stay at a hotel for a procedure that may not be necessary; however, offering a patient monetary incentives for surgery might cloud their judgment.

Similarly, financial incentives to the surgeon(s) or individuals who are carrying out the study would result in major ethical predicaments to patients. The most egregious breach of trust would be for surgeons to advocate this (or any other) procedure to patients in whom the surgery may be contraindicated, in which case the most ethical way to do this surgery would be in the context of a clinical trial until there is proven value of the procedure. For these reasons, it is crucial that no special financial incentives are given to surgeons in the trial. Some concerns were expressed about how soon after the first surgery the re-sampling surgery would be offered. Many patients felt that they would not be psychologically prepared for a second surgery 3 months after the first. They felt that because the time of diagnosis was so chaotic, they did not have time to consider their situation and mourn their diagnosis. At 3 months most patients had finished their surgery, radiation, and chemotherapy. At this point they were beginning to concede the reality of their situation. Patients who had remained calm during the initial diagnosis stated that the realization of what they had been through had just began to dawn on them after 3 months, and at that point if they were asked to undergo a biopsy, they would be psychologically devastated. Some patients, however, had recovered completely within 3 months and were in adequate spirits to be prepared for a second surgery. It is important to assess the emotional state of a patient before asking them to participate in a trial, but it is not often possible to determine exactly how a patient will react. Most patients stated that they would be more comfortable considering a second surgery 6 months after their first.

Patients’ previous experiences also inform their reaction to being asked to participate in a trial surgery. Patients who had participated in clinical trials in the past were more open to the idea. Almost every patient stated that their experiences during the first surgery would be a strong determinant of their willingness to participate in a second surgery. This includes the surgery itself, the recovery process, and their experience with the hospital staff. Almost every patient stated that a complication during the first surgery would affect his or her willingness to consider a second surgery. It is important to keep this in mind when approaching patients about participating in a trial.

Even with all of the concerns that patients expressed, most were interested in a proven re-sampling surgery. Many patients were also interested in the clinical trial testing the value of this procedure. This study suggests that over two thirds of the patients would consider participating in a re-sampling surgery if it were offered. It also suggests that about half of the patients would consider participating in the clinical trial.

### Limitations of this study

The patients sampled were from adult teaching hospitals in a large academic health center in a socialized health care system, being seen at one clinic. These results may not be generalizable to other cities, countries, settings, and cultures.

## Conclusion

It appears that most patients would be interested in resampling surgery for glioblastoma post-therapy or at asymptomatic relapse if it were offered routinely, and many would also be interested in participating in a clinical trial.

Asking patients about the acceptability of a new intervention before it becomes clinically available is not the standard model in current-day health care delivery, but this approach would seem to be logistically and ethically preferable. Finding out whether a “marketplace” exists for new interventions may represent a more responsible way to introduce much-needed innovations in health care, as opposed to the current practice of innovating, and then seeing if there is an application for the innovation that is acceptable to patients.

## Interview questionnaire

### Preamble

This interview is intended to explore your perceptions regarding re-sampling of malignant brain tumour post-therapy and/or at non-symptomatic relapse and to discuss your experiences about the treatments you have undergone so far. Your input is important to us and will help us do a better job in implementing such standards.

### Interview proper

1. Please tell me about how you came to be diagnosed. How did you feel, and what was your initial reaction when your neurosurgeon first diagnosed you with a malignant brain tumour and told you that you needed to have surgery? How were you feeling after that appointment? Tell me more about your experience.
2. We understand that since the discovery of your tumour, it has most likely been a stressful and exhausting time for both you and your family. How have you and your family been coping since? Have you been able to cope well, or have you encountered difficulties along the way? What type(s) of coping mechanisms have you been trying to use to help you through this? Tell me more about it.
3. Your team of doctors has told you about the risks and possible complications associated with brain surgery. Specifically, there is risk of infection, post-operative neurological deficits like speech problems, are or leg weakness, and others. How did you feel when you were being told about these risks? How do you feel about these risks themselves? Are you worried that these things will happen to you? Do you feel scared and/or nervous? Tell me more.
4. What are your thoughts about the brain tumour you currently have? Has this diagnosis affected your feelings about yourself and your attitudes towards life? What short-term and/or long-term goals do you have in life? Tell me more about it.
5. Malignant brain tumours are usually treated with a multiple different treatments and typically involve surgery, radiotherapy, and chemotherapy. What are your thoughts on this, and how do you feel about having to go through these steps? Tell me about it.
6. HYPOTHETICAL CASE SCENARIO: *You have been diagnosed with a glioblastoma (a serious malignant brain tumour with a poor prognosis) and have undergone your first craniotomy for tumour resection about a month ago. Doctors were able to remove part of your tumour, and you are currently undergoing radiotherapy and chemotherapy. You visited your neurosurgeon for a follow-up yesterday afternoon, and he mentions about doing another surgery for you that may help understand your tumour and maybe improve your chances for survival.*
  How do you feel about this? Would you feel eager or reluctant about such an idea? If reluctance is what you feel, why do you think you feel that? Do you think the experiences you had during your last surgery would affect your decision in agreeing to the second surgery? What questions and/or concerns would you express to your doctor at this point?
7. HYPOTHETICAL CASE SCENARIO: *This is a follow-up to the other scenario. Your neurosurgeon informs you about a medical research initiative that may potentially enable doctors to better enhance treatment for brain tumour patients like you. To reach this goal, scientists need to examine, identify, and characterize cancerous cells – those of which we would obtain from the patients. This may or may not benefit you directly, but to have your chances of being benefitted slightly increased, your neurosurgeon must re-operate on you to collect another sample of your initial/recurrent tumour.*
  How would you feel about this? Do you believe an ethical concern has emerged if your doctor recommends you to have surgery again under these circumstances? Would you feel comfortable and proceed with this intervention, or would you feel hesitant and doubtful about it? Alternatively, would you feel obligated to listen to the doctor's instructions because you feel that he or she knows what's best for you (despite your reluctance in doing a second surgery)? Please tell me about your thoughts on this.
8. If you were recommended by your clinician to undergo a repeat surgery (for tumour re-sampling) within 3 months of treatment initiation or at relapse – an operation that could potentially assist him or her in planning for a more effective therapy for you – would you feel comfortable to go ahead with this procedure? Note that it is important to consider the risks of surgery as well. Also, with the restriction of the procedure being done within 3 months of your first surgery – would this be too overwhelming for you? How would you feel? What are the thoughts that come to mind?
9. If you had experienced complications from your initial surgery, and your neurosurgeon suggests you to undergo a repeat surgery (for the same reasons as listed in the previous question), would you agree to do it? Why or why not? Please explain, and tell me more about it.
10. What is your attitude regarding treatment for your diagnosis? Of course, it is expected of you to adhere to the treatment(s) you're required to undergo – after all, your goal is to get better. This process would typically involve several visits to your doctors and require you to spend a fair bit of time at the hospital. If you were to undergo a repeat craniotomy for tumour re-sampling post treatment initiation or at relapse, for instance, you would be required to spend additional time at the hospital. Having said this, we completely understand that you would like to spend more time with family and friends during this difficult period in your life, and because this aspect is important for many people. How willing would you would be to dedicate your time towards experimental medical procedures that may or may not ultimately benefit you. Tell me your thoughts on this.
11. Similar to the bone marrow sampling that doctors do with leukemia patients, we would like to re-sample cancerous brain tumours to gaining a better understanding on glioblastoma evolution with Temozolomide (the chemotherapeutic drug) as well as identifying genetic drivers within a patient's tumour. Ultimately, our goals are to improve patient prognosis, enhance the efficacy of conventional therapies, and to create newly combined treatments that will potentially make patient cancer remissions and cures a more durable process. MRI scans provide only crude information about patients' situations, and we believe that studying tumour genes will allow us to improve the effectives of our current therapies. In order to obtain these brain tumour samples, however, you would need to go for surgery again. Do you think it is ethical to subject patients to repeat sampling after initial treatment? Tell me how you feel about this. Do you feel that going through a procedure like this for the second time will psychologically affect you in any way? How do you think this may affect your family and loved ones? Please explain, tell me more.
12. Is there anything else you would like to add regarding what we just talked about, or in general?

## References

[1] Barami K, Fernandes R (2012). Incidence, risk factors, and management of delayed wound dehiscence after craniotomy for tumor resection. J Clin Neurosci.

[2] Bernstein M, Bampoe J (2004). Surgical innovation or surgical evolution: an ethical and practical guide to handling novel neurosurgical procedures. J Neurosurg.

[3] Bernstein M, Potvin D, Martin DK (2004). A qualitative study of attitudes toward error in patients facing brain tumour surgery. Can J Neuro Sci.

[4] Chakravarti A, Palanichamy K (2008). Overcoming therapeutic resistance in malignant gliomas: current practices and future directions. Rad Oncol Advan.

[5] Chang SM, Parney IF, Mcdermott M, Barker FG, Schmidt MH, Huang W, Laws ER, Lillehei KO, Bernstein M, Brem H, Sloan AE, Berger M, the Glioma Outcomes Investigators (2003). Perioperative complications and neurological outcomes of first and second craniotomies among patients enrolled in the glioma outcome project. J Neurosurg.

[6] Delgado-López PD, Martín-Velasco V, Castilla-Díez JM, Galacho-Harrieroy AM, Rodríguez-Salazar A (2009). Preservation of bone flap after craniotomy infection. Neurocirugia.

[7] Dützmann S, Geßler F, Bink A, Quick J, Franz K, Seifert V, Senft C (2012). Risk of ischemia in glioma surgery: comparison of first and repeat procedures. J Neurooncol.

[8] Fiveash J, Nabors LB (2005). Treatment of adults with recurrent malignant glioma. Expert Rev Neurother.

[9] Kazak AE, Boeving CA, Alderfer MA, Hwang W-T, Reilly A (2005). Posttraumatic stress symptoms during treatment in parents of children with cancer. J Clin Oncol.

[10] Kim SS, McCutcheon IE, Suki D, Weinberg JS, Sawaya R, Lang FF, Ferson D, Heimberger AB, DeMonte F, Prabhu SS (2009). Awake craniotomy for brain tumors near eloquent cortex: correlation of intraoperative cortical mapping with neurological outcomes in 309 consecutive patients. Neurosurgery.

[11] Knifed E, July J, Bernstein M (2008). Neurosurgery patients’ feelings about the role of residents in their care: a qualitative case study. J Neurosurg.

[12] Korinek A-M (1997). Risk factors for neurosurgical site infections after craniotomy: a prospective multicenter study of 2944 patients. Neurosurgery.

[13] Long DM, Gordon T, Bowman H, Etzel A, Burleyson G, Betchen S, Garonzik IM, Brem H (2003). Outcome and cost of craniotomy performed to treat tumors in regional academic referral centers. Neurosurgery.

[14] Mattison RJ, Luger SM, Lazarus HM (2013). New strategies for the evaluation of the nadir bone marrow following induction in acute myeloid leukemia. Curr Opin Hematol.

[15] Patir R, Mahapatra AK, Banerji AK (1992). Risk factors in postoperative neurological infection: a prospective study. Acta Neurochir.

[16] Rooney AG, Carson A, Grant R (2010). Depression in cerebral glioma patients: a systematic review of observational studies. J Natl Cancer Inst.

[17] Schubart JR, Kinzie MB, Farace E (2008). Caring for the brain tumor patient: family caregiver burden and unmet needs. Neuro Oncol.

[18] Serletis D, Bernstein M (2007). Prospective study of awake craniotomy used routinely and nonselectively for supratentorial tumors. J Neurosurg.

[19] Strauss AL, Corbin JM (1990). Basics of qualitative research: grounded theory procedures and techniques.

[20] Stupp R, Mason WP, van den Bent MJ, Weller M, Fisher B, Taphoorn MJB, Belanger K, Brandes AA, Marosi C, Bogdahn U, Curschmann J, Janzer RC, Ludwin SK, Gorlia T, Allegeier A, Lacombe D, Cairncross JG, Eisenhauer E, Mirimanoff RO, for the European Organisation for Research and Treatment of Cancer Brain Tumor and Radiotherapy Groups and the National Cancer Institute of Canada Clinical Trials Group (2005). Radiotherapy plus concomitant and adjuvant temozolomide for glioblastoma. New Engl J Med.

[21] Terasaki M, Ogo E, Fukushima S, Sakata K, Miyagi N, Abe T, Shigemori M (2007). Impact of combination therapy with repeat surgery and temozolomide for recurrent or progressive glioblastoma multiforme: a prospective trial. Surg Neurol.

[22] Verhaak RGW, Hoadley KA, Purdom E, Wang V, Qi Y, Wilkerson MD, Miller CR, Ding L, Golub T, Mesirov JP, Alexe G, Lawrence M, O’Kelly M, Tamayo P, Weir BA, Gabriel S, Winckler W, Gupta S, Jakkula L, Feiler HS, Hodgson JG, James CD, Sarkaria JN, Brennan C, Kahn A, Spellman PT, Wilson RK, Speed TP, Gray JW, Meyerson M, Getz G, Perou CM, Hayes DN, Cancer Genome Atlas Research Network (2010). Integrated genomic analysis identifies clinically relevant subtypes of Glioblastoma characterized by abnormalities in PDGFRA, IDH1, EGFR, and NF1. Canc Cell Art.

[23] Wong J, Bernstein M (2011). Payment of research subjects for more than minimal risk research is unethical. Am J Med Sci.

